# Direct and indirect effects of cathodal cerebellar TDCS on visuomotor adaptation of hand and arm movements

**DOI:** 10.1038/s41598-021-83656-5

**Published:** 2021-02-24

**Authors:** Matthew Weightman, John-Stuart Brittain, R. Chris Miall, Ned Jenkinson

**Affiliations:** 1 School of Sport, Exercise and Rehabilitation Sciences, University of Birmingham, Edgbaston, Birmingham, B15 2TT UK; 2 School of Psychology, University of Birmingham, Edgbaston, Birmingham, B15 2TT UK; 3 MRC-Versus Arthritis Centre for Musculoskeletal Ageing Research, University of Birmingham, Edgbaston, Birmingham, B15 2TT UK; 4 Centre for Human Brain Health, University of Birmingham, Edgbaston, Birmingham, B15 2TT UK

**Keywords:** Cerebellum, Motor cortex, Human behaviour

## Abstract

Adaptation of movements involving the proximal and distal upper-limb can be differentially facilitated by anodal transcranial direct current stimulation (TDCS) over the cerebellum and primary motor cortex (M1). Here, we build on this evidence by demonstrating that cathodal TDCS impairs motor adaptation with a differentiation of the proximal and distal upper-limbs, relative to the site of stimulation. Healthy young adults received M1 or cerebellar cathodal TDCS while making fast ‘shooting’ movements towards targets under 60° rotated visual feedback conditions, using either whole-arm reaching or fine hand and finger movements. As predicted, we found that cathodal cerebellar TDCS resulted in impairment of adaptation of movements with the whole arm compared to M1 and sham groups, which proved significantly different during late adaptation. However, cathodal cerebellar TDCS also significantly enhanced adaptation of hand movements, which may reflect changes in the excitability of the pathway between the cerebellum and M1. We found no evidence for change of adaptation rates using arm or finger movements following cathodal TDCS directly over M1. These results are further evidence to support movement specific effects of TDCS, and highlight how the connectivity and functional organisation of the cerebellum and M1 must be considered when designing TDCS-based therapies.

## Introduction

Recent work from our laboratory demonstrated that anodal transcranial direct current stimulation (TDCS) targeted at the primary motor cortex (M1) or cerebellum selectively improved motor adaptation of distal versus proximal upper-limb movements respectively^1^. Our rationale was derived from the differential level of control M1 and the cerebellum exert over fractionated hand and finger, as opposed to whole-arm movements. M1 is essential for the control and production of independent hand and finger movements^2–4^. Direct damage or lesions to M1 or the corticospinal tract result in severe and permanent deficits in dexterous hand and finger use^5–7^. The importance of the cerebellum in controlling and adapting gross movements of the arm is also well documented^8–13^. Cerebellar output also projects heavily and reciprocally to areas in the parietal cortex known to be important for reaching behaviours and has major connections via the brainstem (e.g. red nucleus) to descending pathways controlling the limbs^14–16^. TDCS is believed to preferentially modulate neural circuits which are active^17–19^. Hence, we predicted (and found^1^) that TDCS would have differential effects on hand vs arm movement, when applied to M1 or the cerebellum.

Notwithstanding those results, M1 and the cerebellum are richly interconnected and interplay between them is critical to dexterous use of the limbs. Both TDCS and transcranial magnetic stimulation (TMS) can influence the excitability of pathways between M1 and the cerebellum^20–22^ and connectivity between these structures is also know to change during motor adaptation and skill learning^23–25^. Given this understanding, we sought to further our investigation of the effector-specific influence of TDCS observed in our previous study^1^, to test whether cathodal stimulation exerts a similarly divergent influence over each of these structures during visuomotor adaptation.

Studies recording motor-evoked potentials or measuring neuronal firing rates have shown that anodal stimulation increases neural excitability of the targeted area whereas cathodal TDCS results in a diminution in excitability^26–28^. However, while there is evidence that both M1 and cerebellar anodal TDCS can enhance motor adaptation^1,29–32^, there is far less or mixed evidence of the behavioural effect of cathodal TDCS. Mechanistically, this has been anecdotally attributed to the conflict between increased levels of neural excitability associated with motor adaptation and the reduction in excitability induced by the cathodal stimulation^17^. Nevertheless, some studies have reported findings showing a decrease in adaptive performance as a result of cathodal TDCS^33–35^. Determining whether direct current stimulation does indeed have polarity specific effects on motor adaptation of proximal and distal movements would inform on the mechanistic interplay of these systems, and guide rehabilitative strategies.

We therefore aimed to combine cathodal TDCS targeting M1 and the cerebellum with motor adaptation tasks that required either hand/finger movements or whole-arm movements^1^. Given the known anatomical connections, and thus bias in activity of the associated circuits, in the context of our previous results, we hypothesised that cathodal cerebellar stimulation would decrease adaptation in a task that predominantly necessitates arm movements, but have no or little effect on a task involving fine-finger/hand movements. In contrast, cathodal TDCS of M1 would impair adaptive performance in the task requiring fine hand/finger movements, and have little to no effect on adaptation involving predominantly arm movements.

## Materials and methods

### Participants

A total of ninety-two healthy right-handed (self-reported) participants were recruited and gave written informed consent to take part in the study (aged 18–26 years, mean = 19.9 ± 1.4 years; 37 females). All methods and protocols were approved and carried out in line with regulations and ethics at the University of Birmingham (Science, Technology, Engineering and Mathematics Ethical Review Committee, University of Birmingham). Participants were all undergraduate students at the University of Birmingham and were offered course credits for participating. Prior to starting the study, safety screening questionnaires for TDCS and TMS were completed. All participants had normal or corrected to normal vision, reported no history of neurological disease and were not taking any centrally acting medication.

Participants were pseudo-randomised into either a whole-arm (vBOT) or hand/finger (joystick) movement task, and then again into one of three stimulation sub-groups: M1 cathodal TDCS, Cerebellar cathodal TDCS or a Sham stimulation group (Table 1). Data from the two sham stimulation sub-groups were the same as reported in our previous study^1^; none of the participants receiving active stimulation had been included in the previous study.

**Table 1:** Summary table of experimental groups.

vBOT task			Joystick task		
M1	Cerebellar	Sham	M1	Cerebellar	Sham
n = 16 (4 females)	n = 15 (6 females)	n = 15 (9 females)	n = 15 (6 females)	n = 16 (5 females)	n = 15 (7 females)

Participants were pseudo-randomly assigned into a whole-arm (vBOT) or hand/finger (joystick) movement task and then sub-grouped into on one three stimulation types (M1, Cerebellar or Sham).

### Apparatus

#### vBOT task

**Figure 1:**
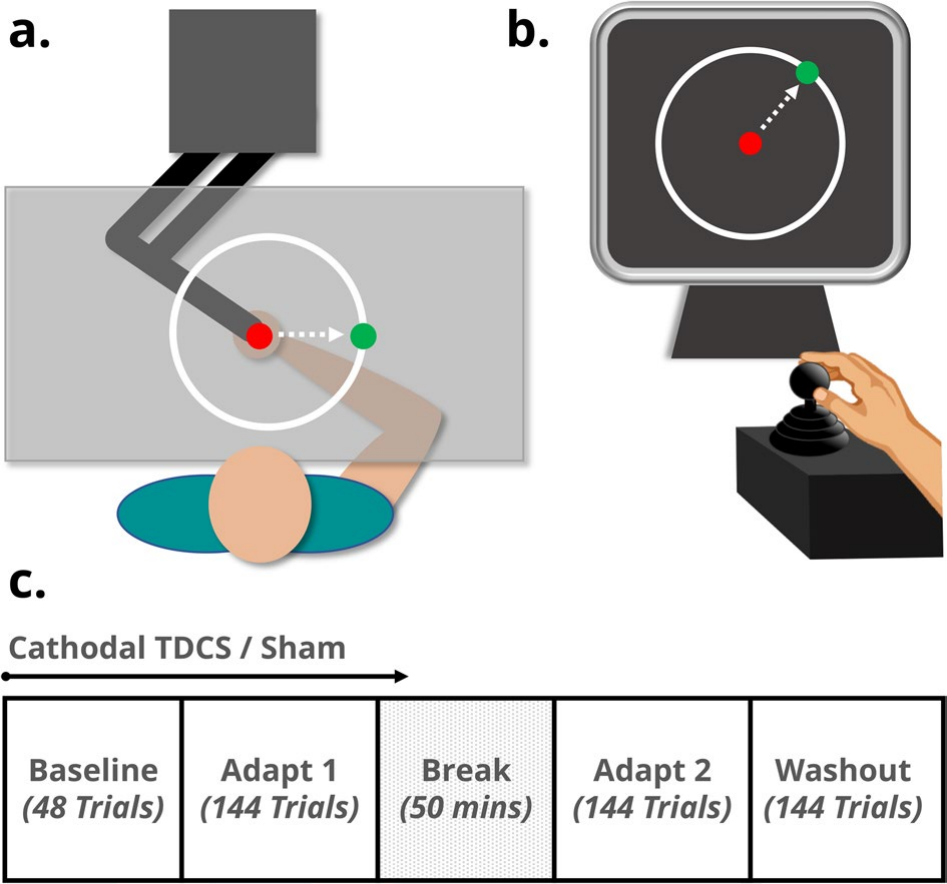
Experimental set up for (**a**) the vBOT task and (**b**) the joystick task, with example screen displays. (**c**). A time course of the study protocol. For each trial, targets would appear at one of eight equidistant locations around the perimeter of the circle, in a pseudorandomised order. In Adapt 1 and Adapt 2, a 60° rotation of the cursor was imposed, with respect to movement of the vBOT handle/joystick. TDCS was started at the beginning of baseline (1.75 min), continued during Adapt 1 (5.25 min) and then was turned off 10 min into the break period. After the break, participants completed Adapt 2 and Washout phases (5.25 min each).

Participants assigned to the vBOT task sat comfortably in front of a custom-built two-dimensional planar robotic manipulandum^36^. With their right hand, they used a power grip to grasp the vertical handle of the manipulandum, which allowed movement in the horizontal plane (Fig. 1a). 10–15 cm movements of the handle were required for the task and were typically achieved via motion of the shoulder and elbow, with limited wrist flexion/extension. The task display was reflected from a computer monitor (Mac Cinema HD Display) onto a horizontal screen (60 × 76 cm) directly below, to appear aligned to the motion of the top of the vBOT handle. Vision of participant’s upper arm was restricted using a curtain attached to the vBOT frame and the room was darkened prior to starting the task. The position of the vBOT handle was measured and digitised at 1000 Hz.

#### Joystick task

For the joystick task, participants sat comfortably in front of a vertical computer monitor (22.5 cm × 30.5 cm) and grasped a small low-profile sprung joystick (APEM 9000 Series, height = 6.5 cm, width = 2 cm) with their index finger and thumb (Fig. 1b). The joystick was located to the right of the computer monitor and was fixed to the desk. 2–3 cm motion of the joystick was attained by index finger and thumb movements with very slight wrist abduction/adduction. Joystick motion was sampled at 75 Hz. An opaque box was used to block vision of participant’s hand and forearm during the task.

### Visuomotor rotation paradigm

The display, stimulus-timings and trial/block protocol of the task were exactly the same for both the vBOT and joystick task. The task display contained a white outline circle (12 cm diameter) which was located in the centre of the screen. Using the vBOT handle or joystick, participants controlled a circular red cursor (0.5 cm diameter). During the task a green target circle (0.5 cm diameter) would ‘jump’ between the centre position of the circle and one of eight equidistant target locations spaced around the perimeter of the circle (Fig. 1a,b). The target had a dwell time of 1 s at each location. Participants were instructed to make fast, straight movements with the vBOT handle/joystick in order to direct the cursor towards and through the target before returning to the central home position. They were asked to avoid making curved movements or correcting the trajectory of their movements once initiated. The position at which the cursor crossed the circle perimeter was marked by an open red circle to indicate participant’s final angle, which remained on screen until the target moved back to the central home position.

The task included baseline trials, two visuomotor adaptation phases (Adapt 1 and Adapt 2) and washout trials (Fig. 1c). During baseline and washout trials participants received veridical cursor feedback, thus the on-screen cursor movement matched that of the vBOT handle/joystick. A 60° counter-clockwise (CCW) rotation was applied to the cursor throughout the two adaptation phases. A 50-min period separated the Adapt 1 and Adapt 2 phases, where the participant remained seated at rest. Prior to the start of the task participants were instructed that at some point a perturbation would be applied to the cursor, however, they were not informed about its nature or when it would be implemented. Participants were instructed to try their best to hit or get as close to the target on every trial and were not told, or asked to employ, any explicit strategies.

### TDCS

2 mA of cathodal TDCS (cTDCS) was delivered through two saline soaked sponge electrodes (5 cm × 7 cm) using a DC-Stimulator (neuroConn, Ilmenau, Germany). For the M1 TDCS group, we used a canonical montage: the cathodal electrode was placed over the hand area of the left motor cortex and the anodal electrode was positioned on the contralateral supraorbital ridge^27,28^. The hand motor ‘hotspot’ was identified in each participant using single pulses of TMS (Magstim Radpid2 stimulator; Magstim Ltd, UK), delivered just above threshold intensity in order to elicit a visible twitch from the first dorsal interosseous muscle. For cerebellar stimulation the cathode electrode was positioned over the right cerebellum, 3 cm lateral to the inion^22,37^ and the anodal electrode attached to the upper aspect of the ipsilateral trapezius muscle. This extra-cephalic electrode montage was used—as in our previous study^1^—in order to maximally stimulate the cerebellar hemisphere and reduce current spread^38,39^. For the sham groups, each participant was pseudo-randomly assigned to either the M1 or cerebellar electrode montage arrangement.

For both the M1 and cerebellar cTDCS groups the current was ramped up over 10 s at the beginning of baseline trials, held at 2 mA for 17 min and then ramped down over 10 s. The stimulation lasted throughout the entirety of baseline, Adapt 1 and 10 min into the break period (Fig. 1c). Sham stimulation was delivered at 2 mA for 30 s at the start of baseline trials with 30 s ramping^40^. After the task participants were asked to rate their levels of perceived comfort and confidence in receiving active stimulation on a ten-point visual analogue scale (VAS), in order to determine the level of blinding achieved.

### Data analysis

Movements of the vBOT handle/joystick were recorded trial-by-trial and analysed using custom MATLAB analysis (version R2018b, Mathworks). The main outcome measure: rotational error, was defined as the angular distance between the target location and the direction of cursor movement at peak velocity. Rotational error calculations were checked trial-by-trial and individual trials were rejected and removed from further analysis if participants failed to follow task instructions e.g. failed to make a movement, made more than one movement, changed their aiming direction mid movement etc. (0.36% of trials were rejected).

Error was averaged every 4 trials into bins to be used for subsequent analysis. Area under the error curve (AUC error) was calculated for each participant in Adapt 1, Adapt 2 and Washout to determine the total rotational error made during each phase of the task. Statistical analyses were performed in MATLAB and SPSS Statistics (IBM, version 26). ANOVAs were carried out in general linear model form; any significant main effects and interactions were followed up with Bonferroni-corrected multiple comparisons (post-hoc tests) and statistical significance was set at *p* < 0.05.

Separate two-way ANOVAs (Task = 2 levels (vBOT/joystick) × Stimulation Group = 3 levels (M1/Cerebellar/Sham TDCS)) were run for each of the four task phases. We do not include phase as a factor in a broader omnibus test (e.g. a 3-way ANOVA) as there are obvious differences between these phases that are simply due to adaptation and after-effect, that would confound the ANOVA. It is only the relative differences between groups at each phase that is of interest.

For clarity we initially report the results from each task separately with regards to adaptation, however these were run in a single two-way ANOVA in order to avoid accruing type I errors.

## Results

### No differences between groups in baseline performance

**Figure 2:**
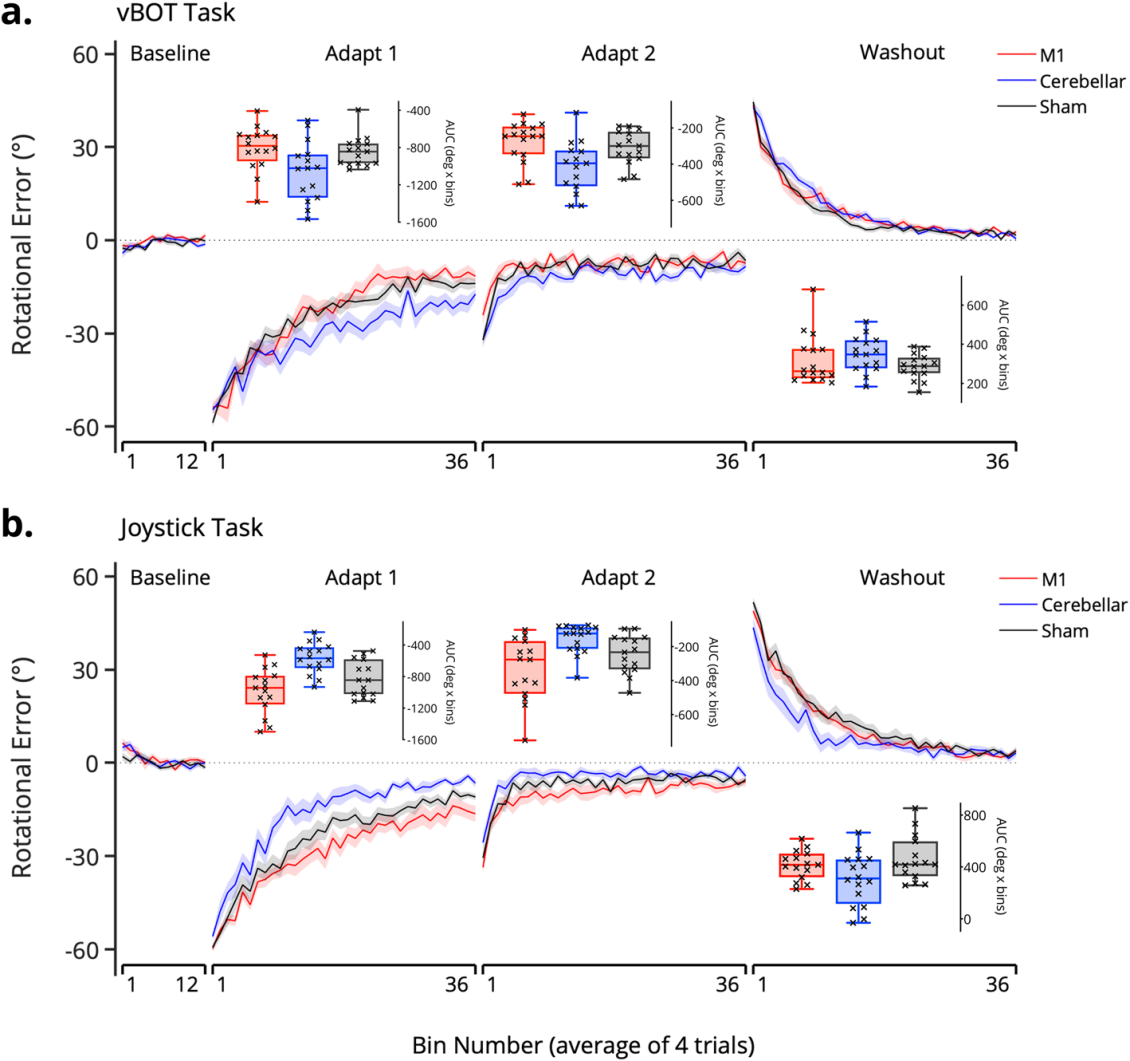
Mean rotational error curves (error over bins of 4 trials, ± standard error = shaded region) for (**a**) the vBOT task and (**b**) the joystick task. Box plot inserts depict the average area under the curve (AUC error, degrees x bins) for each group in the three adaptation phases, with individual data points marked by an ‘x’ (the box indicates mean, upper and lower quartiles; the range shown by the error bars).

Mean error in the last six bins of the baseline phase was compared across the experimental groups. The two-way ANOVA (Task × Stimulation Group) revealed no significance main effects for Task (F(1, 86) = 0.44, *p* = 0.51, ηp^2^ = 0.005) or Stimulation Group (F(2, 86) = 3.07, *p* = 0.052, ηp^2^ = 0.067) and no significant Task*Stimulation Group interaction (F(2, 86) = 0.41, *p* = 0.67, ηp^2^ = 0.009). These results suggest that all participants were performing similarly towards the end of the baseline (Fig. 2), making certain that any differences during the adaptation phases were not due to differences in baseline performance.

### Adaptation and de-adaptation performance varied between the two tasks

Two-way ANOVAs revealed a significant main effect of Task in each of the Adapt 1, Adapt 2 and washout phases (Adapt 1: F(1, 86) = 4.27, *p* = 0.042, ηp^2^ = 0.047, Adapt 2: F(1, 86) = 11.05, *p* = 0.001, ηp^2^ = 0.14 and washout: F(1, 86) = 7.03, *p* = 0.01, ηp^2^ = 0.076). Comparisons showed that participants in the joystick task made less error during Adapt 1 and 2 compared to the vBOT task, whereas they made greater error during washout trials, suggesting that the vBOT version of the task is harder to learn, and has less after-effect. However, as we never directly compare adaptation between the two tasks this difference does not present a confound when analysing results further.

### Cerebellar cTDCS decreased adaptation in the vBOT task

During Adapt 1 cathodal cerebellar TDCS decreased adaptive performance in the vBOT task compared to the M1 and sham group (Fig. 2a). Post-hoc tests on AUC error (Bonferroni corrected for multiple comparisons) following a significant Task*Stimulation Group interaction; F(2, 86) = 15.33, *p* < 0.001, ηp^2^ = 0.26, revealed this trend was significant between the cerebellar and M1 group (*p* = 0.019) while a strong trend was found for the cerebellar and sham group difference (*p* = 0.059). There was no significant difference between AUC error for the M1 and sham groups (*p* = 1.00).

A two-way ANOVA also revealed a significant Task*Stimulation Group interaction during Adapt 2 (F(2, 86) = 13.001, *p* < 0.001, ηp^2^ = 0.23). Post-hoc tests showed that the cerebellar group continued to make significantly more error than the M1 group (*p* < 0.009), with again a trend between cerebellar and sham AUC errors (*p* = 0.072) and no difference between M1 and sham (*p* = 1.00).

During washout, despite a significant Task*Stimulation Group interaction; F(2, 86) = 4.97, *p* = 0.009, ηp^2^ = 0.1, there were no differences in AUC error between any of the stimulation groups (all *p* > 0.63). These findings suggest that all participants in the vBOT task made a similar amount of errors during washout, likely stemming from them reaching comparable levels of adaptation at the end of Adapt 2. The significant interaction was driven by differences in washout performance in the joystick task (next section).

### Cerebellar cTDCS improved adaptation in the joystick task

Contrary to our hypothesis for the joystick task, participants in the cerebellar group displayed significantly less error during Adapt 1 than the M1 and sham groups (post-hoc *t* tests: *p* < 0.01 and *p* = 0.009 respectively, following a significant Task*Stimulation Group interaction from the two-way ANOVA, F(2, 86) = 15.33, *p* < 0.001, ηp^2^ = 0.26). Despite a slight decrease in performance compared to the sham group (Fig. 2b), the effect of cathodal M1 stimulation was not statistically significant (*p* = 0.28).

Post-hoc tests following the significant Task*Stimulation Group interaction (F(2, 86) = 13.001, *p* < 0.001, ηp^2^ = 0.23), showed at the cerebellar group continued to make less error than the M1 group during Adapt 2 (*p* < 0.001), with no other differences between the M1 and sham group (*p* = 0.15) or cerebellar and sham group (*p* = 0.13).

For washout trials, post-hoc tests following a significant Task*Stimulation Group interaction, F(2, 86) = 4.97, *p* = 0.009, ηp^2^ = 0.1, showed no differences in de-adaptation between the M1 and either the sham (*p* = 0.084) or cerebellar (*p* = 1.00) groups. There was however, a significant difference between the cerebellar and sham groups (*p* = 0.007), as participants in the cerebellar group made less error during de-adaptation (Fig. 2b).

### Exploratory analyses of adaptation time-course

As cerebellar cTDCS produced significant behavioural changes when analysed at the task-phase level, we performed exploratory analyses to further understand how differences in error between the stimulation groups evolved over time. Each of the adaptation and washout phases were split into three equal sections and the AUC error calculated for each third. These sub-phase errors (for the 1st, 2nd and 3rd area under the curve sections) were analysed separately for each task phase using two-way ANOVAs (Task*Stimulation Group).

**Figure 3:**
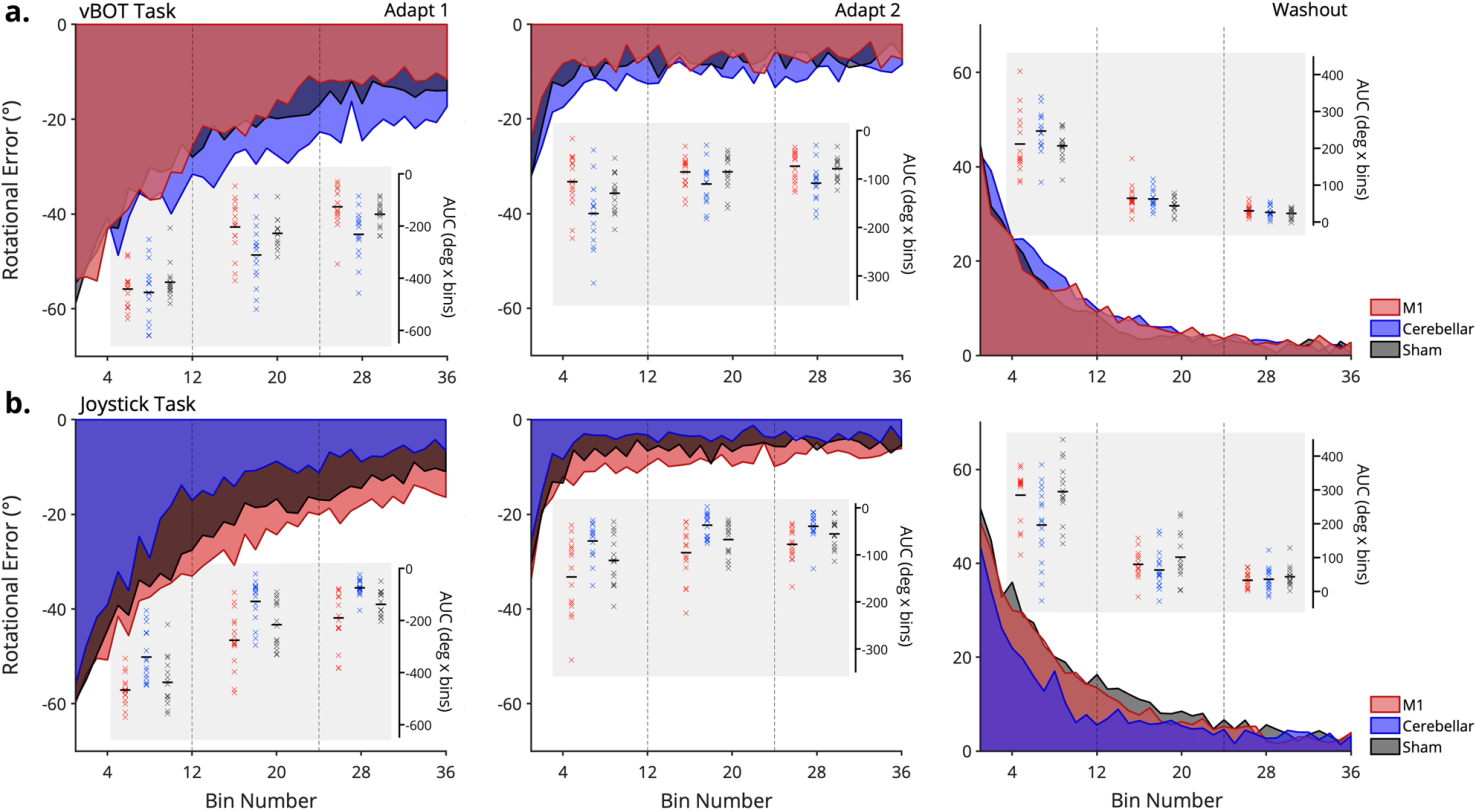
Visualisations of the area under the curve (AUC; degrees x bins) for each adaptation phase in (**a**) the vBOT task and (**b**) joystick task. Scatter plot inserts represent the individual data for each participant in each section of the task phase, with group means shown as short black dashes. Vertical dashed lines signify where the overall data is split into the 3 equal sections.

During Adapt 1 there was a significant Task*Stimulation Group interaction for all 3 sub-sections of the adaptation phase (1st section: F(2, 86) = 6.3, *p* = 0.003, ηp^2^ = 0.13, 2nd section: F(2, 86) = 14.2, *p* < 0.001, ηp^2^ = 0.25 and 3rd section: F(2, 86) = 17.93, *p* < 0.001, ηp^2^ = 0.29). Post-hoc tests revealed that there were no significant differences between the three stimulation groups in the vBOT task during the first section of Adapt 1 (all *p* > 0.69), suggesting that all groups performed similarly during early adaptation (Fig. 3a). Conversely, for the joystick task, post-hoc tests showed that the cerebellar group displayed significantly less error than both the M1 and sham groups during the first section (*p* < 0.001 and *p* = 0.009 respectively), with no differences between the M1 and sham groups (*p* = 1.00), see Fig. 3b. For the second section of Adapt 1, in the vBOT task, cathodal cerebellar TDCS blunted adaptation performance significantly compared to the M1 group (*p* = 0.008), with a near significant decrease compared to the sham group also found (*p* = 0.063). There remained no significant differences between performance in the M1 and sham groups, *p* = 1.00. For the joystick task, the cerebellar group continued to display significantly less error than the M1 (*p* < 0.001) and sham (*p* = 0.035) groups, with no differences between the latter two groups (*p* = 0.28). In the third section of Adapt 1, participants in the cerebellar group made significantly more error than both the M1 (*p* < 0.001) and sham (*p* = 0.016) groups, suggesting cathodal cerebellar TDCS had a greater effect on the final adapted level in the vBOT task. As adaptation started to plateau in the joystick task, post-hoc test revealed that there was only a difference between the cerebellar and M1 groups (*p* < 0.001), with the cerebellar group making significantly less error. The mean difference in area under the curve between the sham and cerebellar group was 63.3 (degrees × bins), but this failed to reach statistical significance (*p* = 0.056). There was again no difference between M1 and sham groups, p = 0.16. In summary, exploratory analysis revealed that cathodal cerebellar TDCS impaired late adaptation to a greater extent during Adapt 1 in the vBOT task. Whereas for the joystick task, it resulted in a greater reduction of error during early and middle adaptation.

Repeating this analysis for the sub-sections within Adapt 2 revealed a significant Task*Stimulation Group interaction for the first section; F(2, 86) = 11.54, *p* < 0.001, ηp^2^ = 0.21, second section; F(2, 86) = 8.81, *p* < 0.001, ηp^2^ = 0.17 and third section; F(2, 86) = 10.55, *p* < 0.001, ηp^2^ = 0.19. Post-hoc tests for the first section of Adapt 2 showed that there was only a significant difference between the M1 and cerebellar groups in both the vBOT and joystick task (*p* = 0.007 and *p* = 0.001 respectively), with the cerebellar group making significantly more error in the vBOT task and significantly less error in the joystick task. All other comparisons were non-significant, *p* > 0.16 (Fig. 3). For the second section, there were no differences in adaptation between any of the stimulation groups in the vBOT task (all *p* > 0.26). In the joystick task the cerebellar group continued to make significantly less error than the M1 group (*p* < 0.001), with no differences between any of the other groups (M1 vs Sham: *p* = 0.18, Cerebellar vs Sham: *p* = 0.103). Post-hocs for the third section of Adapt 2 showed that, similarly to Adapt 1, participants in the cerebellar group made significantly more error at the end of the adaptation phase compared to M1 and sham groups (*p* = 0.008 and *p* = 0.036 respectively) in the vBOT task. As in the previous sections of Adapt 2, there was only a significant difference in adaptation performance between cerebellar and M1 groups (*p* = 0.004) in the joystick task. All other groups performed similarly, *p* > 0.18. Further analyses of Adapt 2 showed relatively inconclusive results, however there appears to be a clear trend of reduced error following cathodal cerebellar TDCS in the joystick task.

Finally, during washout trials a two-way ANOVA revealed a significant Task*Stimulation Group interaction for the first and second sections of washout (1st section: F(2, 86) = 6.67, *p* = 0.002, ηp^2^ = 0.13; 2nd section: = 3.17, *p* = 0.047, ηp2 = 0.069), but not the third section: F(2, 86) = 1.09, *p* = 0.34, ηp^2^ = 0.025). Post-hoc tests showed that the cerebellar group in the joystick task displayed significantly less error during the first third of washout trials than the M1 (*p* = 0.011) and sham (*p* = 0.004) groups. This result suggests that participants were able to reduce error to a greater extent early in de-adaption following cathodal cerebellar TDCS (Fig. 3b). There were no other significant differences between stimulation groups for either the joystick task or vBOT task for any of the other sections of washout (*p* < 0.065), suggesting that de-adaptation occurred relatively similarly across groups regardless of stimulation.

### Stimulation ratings

All participants rated relatively low levels of discomfort (vBOT task means: M1 = 3.06; Cerebellar = 3.0; Sham = 3.53, Joystick task means: M1 = 2.87; Cerebellar = 3.44; Sham = 3.13) on the 10-point VAS (1 = no discomfort, 10 = very high discomfort). A two-way ANOVA (Task*Stimulation Group) revealed there were no significant differences in participant’s rating of comfort during the task. The main effects of Task and Stimulation Group were non-significant (Task: F(1, 86) = 0.061, *p* = 0.81, ηp^2^ = 0.001, Stimulation Group: F(2, 86) = 0.45, *p* = 0.64, ηp^2^ = 0.01) and no significant Task*Stimulation Group interaction (F(2, 86) = 0.78, *p* = 0.46, ηp^2^ = 0.02).

Likewise, participants in all three stimulation conditions reported reasonably high levels of confidence on the 10-point VAS (1 = low confidence, 10 = very confident) that they received active TDCS rather than sham/placebo stimulation (vBOT task means: M1 = 7.69; Cerebellar = 7.53; Sham = 7.8, Joystick task means: M1 = 8.13; Cerebellar = 8.31; Sham = 7.87). There was no main effect of Task (F(1, 86) = 1.9, *p* = 0.17, ηp^2^ = 0.022) or Stimulation Group (F(2, 86) = 0.032, *p* = 0.97, ηp^2^ = 0.064) and no interaction of these terms (F(2, 86) = 0.43, *p* = 0.65, ηp^2^ = 0.01). This result suggests that effective blinding of the stimulation condition was achieved.

## Discussion

In this study we aimed to differentially disrupt motor adaptation of movements made with either the whole arm or the hand, by inhibiting M1 or cerebellar function with cathodal TDCS (cTDCS). Based on previous work in our lab, the physiology of motor systems, and the proposed activity-selective effects of stimulation, we hypothesised that cerebellar cTDCS would impair adaptation of arm movements mainly involving the proximal shoulder and elbow joints, whereas M1 cTDCS would impair adaptive performance using fine hand and finger movements.

Indeed, when compared across the experimental phases, cerebellar cTDCS significantly impaired motor adaptation of the proximal upper-limb compared to M1 stimulation, with a near-significant impairment compared to sham (*p* = 0.056). This latter difference became more obvious with time, and an exploratory analysis indicated that by the end of the first phase, the cerebellar effect was also significantly different from the sham group. Thus, participants who received cathodal cerebellar stimulation continued to make large errors, while both the sham and M1 groups approached a lower error asymptote. As the cerebellum is known to be crucial in the control and adaptation of arm movements^9,11^, reducing its excitability via cTDCS is likely to compromise adaptive performance when using whole-arm reaching movements, as displayed here. Herzfeld and colleagues^41^ likewise reported an impairment of performance in a force field reaching task following cerebellar cTDCS, while Jayaram et al.^33^ have shown disrupted gait adaptation. Depressing cerebellar excitability with repetitive TMS has also been shown to impair cerebellar-dependent adaptation of saccadic eye movements^42^. It is also reported that increasing cerebellar excitability using anodal TDCS enhances adaptation of whole-arm reaching movements^1,29,30,37^, complementing our present results. Additionally, patients with cerebellar impairments often display severe deficits in the accuracy and control of whole-arm reaching behaviour and adaptation^10,43,44^. There is however, a current debate surrounding the effectiveness of TDCS on motor behaviour, with an increasing number of studies reporting null effects or failing to replicate previous results^45–48^. We believe that the results presented here, coupled with our previous findings^1^, suggest that TDCS is very sensitive to a combination of stimulation target site and task demands; specifically, regarding biases in activity of neural circuits associated with the particular movements required.

As M1 dominates the control of dexterous hand movements^4^ and previous research in our lab has shown that anodal TDCS increases adaptation performance when the task requires fine hand and finger movements^1,31^, we predicted that cTDCS of M1 would impair performance in the joystick task. Contrary to this hypothesis, despite a trend towards lower levels of error reduction, there were no significant differences between the M1 and sham group at any point during the task. This result is not out of place within the extant literature on M1 cTDCS and motor skill learning/performance^22,49,50^. One possible explanation for the lack of effect is competition between increased M1 excitability associated with motor learning^51,52^ and the depressive effects of cTDCS^17^, thus preventing significant change in performance. Given that all participants in the current study were healthy young adults, floor effects may also have had some bearing on the results, especially as adaptation performance was more complete in the joystick task compared to the vBOT task.

However, we also observed an unexpected enhancement of motor adaptation in the joystick task following cerebellar cTDCS, compared to both M1 and sham stimulation. Thus, cerebellar cTDCS impaired adaptation of the arm, and enhanced adaptation of the hand and fingers. Although not hypothesised, there is a clear physiological foundation for the latter effect when considering the connectivity between M1 and the cerebellum. As discussed previously, increased excitability of M1 can improve motor learning and adaptation, particularly when using the hand/fingers^1,31,49^. It is well established that there are functional connections between the cerebellum and M1 (see review Allen and Tsukahara^53^). Purkinje cells, which are the sole output neurons of the cerebellar cortex, have inhibitory connections with the dentate nucleus (the largest of the deep cerebellar nuclei), which in turn projects to the motor cortex via disynaptic excitatory pathways through the thalamus. These complex connections form the cerebello-thalamo-cortical pathway, one of the major output systems of the cerebellum^53–55^. At rest this pathway exerts an overall inhibitory tone over the motor cortex, termed cerebello-brain inhibition (CBI). It is believed that TDCS modulates Purkinje cell excitability to either increase or decrease CBI, depending of the polarity of stimulation used^22,27^. For example, Ugawa and colleagues^56^ applied anodal electrical currents across the cerebellum, which resulted in short-lasting inhibition of the contralateral M1 (i.e. an increase in CBI). More recently, Galea et al.^22^ showed that modulation of M1-cerebellar connectivity is polarity specific: anodal cerebellar TDCS enhanced CBI, whereas cathodal TDCS decreased it, disinhibiting M1 and therefore increasing excitability. Given these findings, we speculate that cerebellar cTDCS increased M1 excitability and thus indirectly facilitated adaptation of fine hand and finger movements.

Many studies have investigated the relationship between M1 and the cerebellum during motor adaptation and skill learning^23–25,57,58^. But to our knowledge no study has reported a significant motor effect linked to CBI modulation (see Pope and Miall^59,60^, for a related cognitive effect). Further research must be conducted in order to mechanistically link the behavioural effect displayed here to a decrease in CBI. It may be particularly interesting to probe the excitability of the motor system with TMS, pre and post intervention, in order to better understand the interaction between TDCS induced changes in neural excitability of different brain regions and motor learning. The capability to improve hand and finger use via stimulation of a brain area distant from M1 may have important practical implications for TDCS use in rehabilitation. Especially when direct targeting of an injured brain area—following stroke or traumatic brain injury etc.—may be best avoided due to contraindications, such as inducing seizures.

Recently, it has become clear that both implicit and explicit learning processes contribute to successful adaptive behaviour^61–65^. Implicit processes drive gradual adaptation to small discrepancies between predicted and actual consequences of movements, by updating internal models of the imposed perturbation (see review Shadmehr et al.^12^). Implicit sensorimotor adaptation is cerebellar dependent, and thought to constitute the slow process of adaptation^11,66^. Explicit processes on the other hand, allow a strategic component to the adaptation of large and sudden perturbations. Learners detect the perturbation and consciously select appropriate actions in order to counter it^67,68^. Explicit processes are believed to be more involved during the fast process of adaptation and to be under greater cortical control^63^. Results from the present study, especially our exploratory analysis of the time course of the adaptation phases, suggest that cerebellar cTDCS impairs late adaptation to a greater extent in the vBOT task and promotes error reduction early in the joystick task. Given that we suggest the increased performance in the joystick task is the result of CBI-driven disinhibition of the neocortex, and that TDCS of the cerebellum is not focal, one could argue that the cerebellar cTDCS may have facilitated frontal areas outside of M1^59^. The prefrontal cortex is classically associated with the explicit component of sensorimotor adaptation, due to its role in cognitive control and working memory^69,70^. In contrast, cathodal cerebellar stimulation impaired performance during late adaptation in the vBOT task, which may reflect a disruption of the direct cerebellar dependent slower process of learning (although the cerebellar contribution to motor learning is not solely confined to the latter stages^24,71^). However, as we did not specifically aim to probe the various processes of motor adaptation, these conclusions must be only speculative.

**Figure 4:**
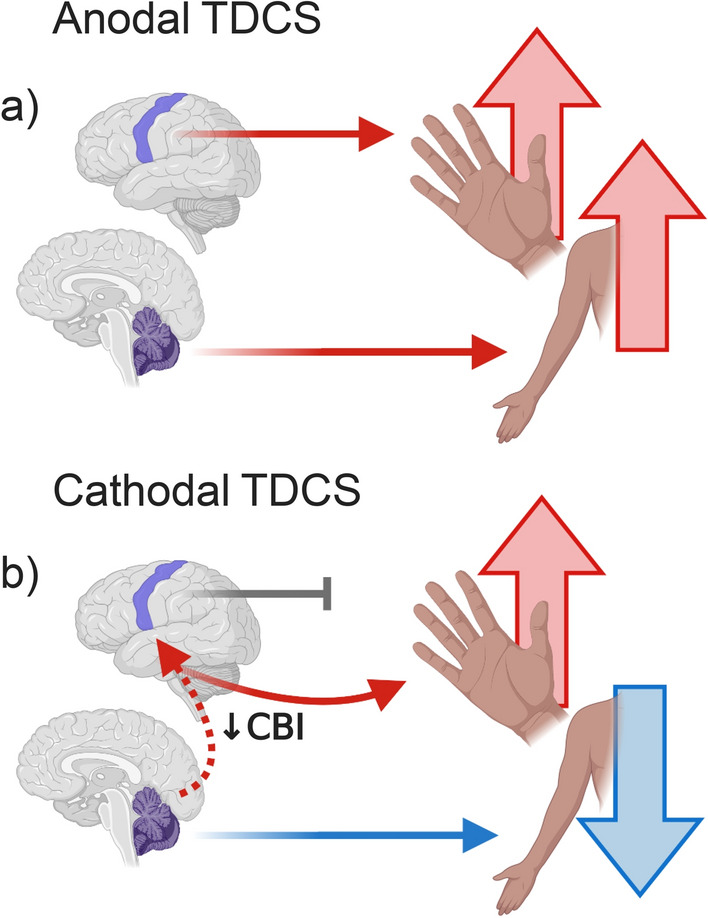
A schematic diagram of the proposed framework of TDCS effects, devised from current and previous results. (**a**) The dissociated effects of anodal TDCS on upper-limb motor adaptation reported in Weightman et al.^1^, whereby M1 TDCS selectively enhanced adaptation of dexterous hand movements and cerebellar stimulation enhanced adaptation of whole-arm reaches. (**b**) Portrays results from the present study. As hypothesised cathodal cerebellar TDCS impaired adaptation of reaching movements, however with no significant effect of M1 cathodal TDCS on hand and finger movements. We also found cathodal cerebellar TDCS indirectly improved adaptation of dexterous hand movements, which we suggest to be a result of disinhibition of M1 via a reduction in cerebello-brain inhibition (CBI). *Created with BioRender.com*.

In summary, we have shown that adaptation of arm reaching movement is impaired following cathodal cerebellar stimulation, consistent with our previous work that argued the cerebellum was more responsible for adaptation of movements involving the proximal musculature, whereas M1 was more involved in adaptation of distal hand/finger movements. Paradoxically, then, we also found that fine hand and finger adaptation was enhanced by cathodal cerebellar TDCS, and we propose this is due to disinhibition of M1 via a reduction in CBI (Fig. 4). In other words, we suggest a direct effect of cerebellar cTDCS on proximal control and an indirect effect on distal control. We found no evidence however, for a significant effect of cathodal M1 TDCS on either arm or hand adaptation, although there was a non-significant and weak trend towards reduced adaptation in the hand task, suggesting a single-session of stimulation using these parameters in healthy individuals had null effects. Our results provide additional evidence to support an emerging framework, which suggests TDCS affects neural circuits in an activity-specific manner, consistent with a bias in activity within the motor hierarchy for control of proximal and distal movement.
